# 2-[2-(Trifluoro­meth­yl)phen­yl]-2*H*-1-benzopyran-4(3*H*)-one

**DOI:** 10.1107/S160053681201687X

**Published:** 2012-04-25

**Authors:** Bojana M. Francuski, Branka Ivković, Ivana Stojanović, Sote Vladimirov, Djordje Francuski

**Affiliations:** a VINČA Institute of Nuclear Sciences, Laboratory of Theoretical Physics and Condensed Matter Physics, University of Belgrade, PO Box 522, 11001 Belgrade, Serbia; b Department of Pharmaceutical Chemistry, Faculty of Pharmacy, University of Belgrade, Vojvode Stepe 450, 11010 Belgrade, Serbia; c Institute of Molecular Genetics and Genetic Engineering, University of Belgrade, Vojvode Stepe 444a, 11010 Belgrade, Serbia

## Abstract

In the title compound, C_16_H_11_F_3_O_2_, the γ-pyran­one ring adopts an envelope conformation with the chiral C atom standing out of the ring plane. In the crystal, molecules are linked by C—H⋯O and C—H⋯F inter­actions.

## Related literature

For general background to flavones, see: Harborne & Williams (2000 ▶). For related flavonoids, see: Benavente-García & Castillo (2008 ▶); Rodeiro *et al.* (2006 ▶). For related structures, see: Wera *et al.* (2012 ▶); Białońska *et al.* (2007 ▶); Krishnaiah *et al.* (2005 ▶); Wu *et al.* (2005 ▶). For van der Waals radii, see: Bondi (1964 ▶).
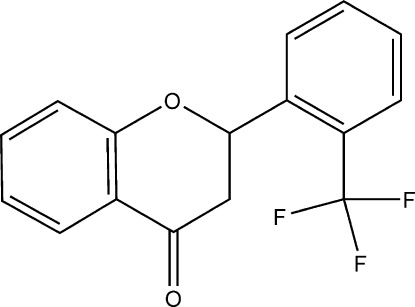


## Experimental

### 

#### Crystal data

C_16_H_11_F_3_O_2_
*M* *_r_* = 292.25
Orthorhombic, 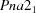

*a* = 8.2291 (9) Å
*b* = 22.020 (3) Å
*c* = 7.3355 (11) Å
*V* = 1329.2 (3) Å^3^
*Z* = 4
Mo *K*α radiation
μ = 0.12 mm^−1^
*T* = 293 K
0.18 × 0.02 × 0.02 mm

#### Data collection

Oxford Diffraction Xcalibur Sapphire3 Gemini diffractometer
4585 measured reflections
2558 independent reflections
1462 reflections with *I* > 2σ(*I*)
*R*_int_ = 0.049

#### Refinement

*R*[*F* ^2^ > 2σ(*F* ^2^)] = 0.087
*wR*(*F* ^2^) = 0.109
*S* = 1.12
2558 reflections
190 parameters
1 restraint
H-atom parameters constrained
Δρ_max_ = 0.16 e Å^−3^
Δρ_min_ = −0.15 e Å^−3^

### 

Data collection: *CrysAlis PRO* (Oxford Diffraction, 2010 ▶); cell refinement: *CrysAlis PRO*; data reduction: *CrysAlis PRO*; program(s) used to solve structure: *SHELXS97* (Sheldrick, 2008 ▶); program(s) used to refine structure: *SHELXL97* (Sheldrick, 2008 ▶); molecular graphics: *ORTEP-3* (Farrugia, 1997 ▶); software used to prepare material for publication: *WinGX* (Farrugia, 1999 ▶), *PLATON* (Spek, 2009 ▶) and *PARST* (Nardelli, 1995 ▶).

## Supplementary Material

Crystal structure: contains datablock(s) I, global. DOI: 10.1107/S160053681201687X/kj2198sup1.cif

Structure factors: contains datablock(s) I. DOI: 10.1107/S160053681201687X/kj2198Isup2.hkl

Supplementary material file. DOI: 10.1107/S160053681201687X/kj2198Isup3.mol

Supplementary material file. DOI: 10.1107/S160053681201687X/kj2198Isup4.cml

Additional supplementary materials:  crystallographic information; 3D view; checkCIF report

## Figures and Tables

**Table 1:** Hydrogen-bond geometry (Å, °)

*D*—H⋯*A*	*D*—H	H⋯*A*	*D*⋯*A*	*D*—H⋯*A*
C3—H3⋯O2^i^	0.93	2.54	3.311 (5)	140
C5—H5⋯F3^ii^	0.93	2.54	3.313 (5)	141

Symmetry codes: (i) 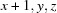; (ii) 
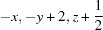
.

**Fig. 1.**
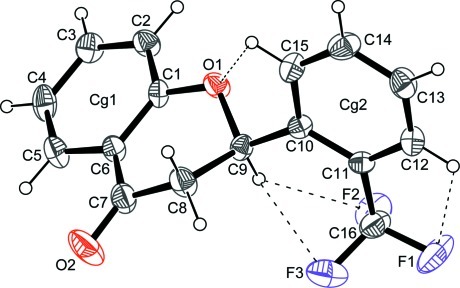
The molecular structure of (I), with atom labels and 30% probability displacement ellipsoids for non–H atoms. H atoms are represented as small spheres of arbitrary radii. Intramolecular interactions are shown as dashed lines.

**Fig. 2.**
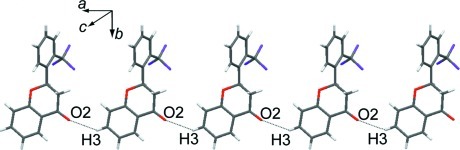
Molecular packing of the title compound and intermolecular interactions along the crystallographic a axis.

**Fig. 3.**
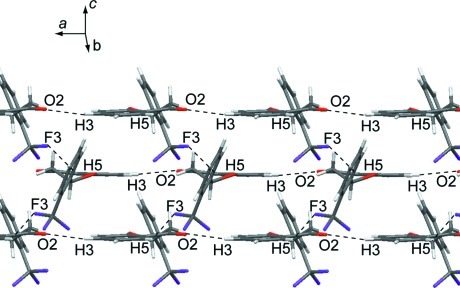
Molecular packing of the title compound and intermolecular interactions along the crystallographic c axis.

**Fig. 4.**
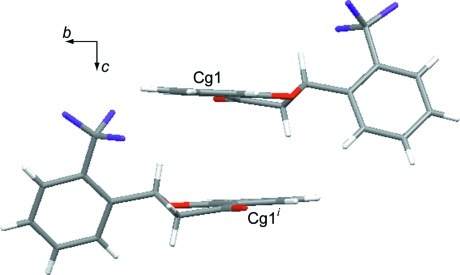
π–π interactions of two neighbouring Cg1 rings. Symmetry code: (i) 1 - x, 2 - y, 1/2 + z.

## supplementary crystallographic information

### Comment

The title compound belongs to the group of flavanones which occur predominantly in citrus fruits. Citrus flavonoids were reported (Benavente-García & Castillo, 2008) as having antimicrobial, antifungal, antiviral, anti–allergenic, anti-inflammatory (Harborne & Williams, 2000) and antioxidant (Rodeiro *et al.*, 2006) properties. Case control studies suggest that flavonoids may reduce the risk of cardiovascular disease and stroke. Considering the wide spectrum of activities of natural flavonoids further structure modification of these molecules is aimed to enhance their interaction with the target sites in cells.

The title compound adopts a typical conformation of flavanones with the γ–pyranone ring adopting the envelope conformation. In this conformation the carbon atoms C1, C6, C7, C8 and oxygen O1 are nearly coplanar with a root mean square deviation from the mean plane of 0.035 Å, while the C9 carbon atom is standing out from this plane with an atom–to–plane distance of 0.652 (6) Å. All bond lengths and angles in the title compound show usual values for this type of compounds (Wera *et al.*, 2012; Białońska *et al.*, 2007; Krishnaiah *et al.*, 2005; Wu *et al.*, 2005). In this crystal packing of the title compound there are 4 intramolecular, 2 intermolecular (C—H···O and C—H···F) and one π–π interaction. All of the fluoride atoms participate in weak intramolecular C—H···F interactions (Fig. 1) with H···F distances equal or shorter than 2.50 Å, which is shorter than the sum of their Van der Waals radii (Bondi, 1964). Two of the fluoride atoms interact with the hydrogen atom connected to the chiral carbon atom C9 while the third fluoride atom interacts with one of the phenyl hydrogen atoms (Table 1). The dihedral angle between *Cg*1 and *Cg*2 rings in previously published structures of flavanones (Wera *et al.*, 2012; Białońska *et al.*, 2007; Krishnaiah *et al.*, 2005; Wu *et al.*, 2005) is in the range from 55 to 75° while the corresponding dihedral angle in the case of the title compound is 66.06 (15)°.

The flavanone molecules are connected into rows by the C3—H3···O2 intermolecular interaction forming chains down the crystallographic *a* axis (Fig. 2). The second intermolecular interaction, C5—H5···F3, connects the molecules into another chain in the direction of the screw axis following the crystallographic *c* axis, thus forming a two dimensional net of molecules (Fig. 3). Strings of molecules along the crystallographic *c* axis are further connected by π–π interactions (Fig. 4). Perpendicular distance from the centroid of one *Cg*1 ring, molecule at (*x*, *y*, *z*), to the plane of the second *Cg*1 ring, molecule at (1 - *x*, 2 - *y*, 1/2 + *z*), and *vice versa* are 3.70 and 3.62 Å respectively, while the distance between the ring centroids measures 4.101 (3) Å. The *Cg*1 ring planes of molecules at (*x*, *y*, *z*) and (1 - *x*, 2 - *y*, 1/2 + *z*) are nearly parallel with the dihedral angle being 5.59 (3)°.

### Experimental

The proposed compound was prepared in two steps. The first step in the synthetic route consisted of the condensation of 2-hydroxy acetophenone with 2-trifluoromethylbenzaldehyde to give an α,β-unsaturated ketone (chalcone). In the second step the obtained chalcone was dissolved under stirring in a 50:50 water/ethanol mixture. The pH was set to 9.0 with 0.1 *M* NaOH and the reaction mixture was refluxed for 2 h after which it was cooled over night to room temperature. Small crystals of the title compound formed in the reaction vessel and, after draining excess fluid, the crystals were dried at room temperature.

### Refinement

The H atoms bonded to C atoms were placed at geometrically calculated positions and refined using a riding model. C—H distances were fixed to 0.93 Å for aromatic C atoms, 0.97 Å for the secondary CH_2_ group and 0.98 Å for the tertiary CH group. Their *U*_iso_(H) values were equal to 1.2 times *U*_eq_ of the corresponding C atom.

In the absence of significant anomalous scattering, the absolute configuration could not be reliably determined so the Friedel pairs were merged and any references to the Flack parameter were removed.

### Crystal data

**Table:**

C_16_H_11_F_3_O_2_	*F*(000) = 600
*M**_r_* = 292.25	*D*_x_ = 1.460 Mg m^−^^3^
Orthorhombic, *P**n**a*2_1_	Mo *K*α radiation, λ = 0.71073 Å
Hall symbol: P 2c -2n	Cell parameters from 966 reflections
*a* = 8.2291 (9) Å	θ = 3.3–28.9°
*b* = 22.020 (3) Å	µ = 0.12 mm^−^^1^
*c* = 7.3355 (11) Å	*T* = 293 K
*V* = 1329.2 (3) Å^3^	Needle, colourless
*Z* = 4	0.18 × 0.02 × 0.02 mm

### Data collection

**Table:**

Oxford Diffraction Xcalibur Sapphire3 Gemini diffractometer	1462 reflections with *I* > 2σ(*I*)
Radiation source: Enhance (Mo) X-ray Source	*R*_int_ = 0.049
Graphite monochromator	θ_max_ = 28.9°, θ_min_ = 3.7°
Detector resolution: 16.3280 pixels mm^-1^	*h* = −11→10
ω scans	*k* = −28→29
4585 measured reflections	*l* = −9→9
2558 independent reflections	

### Refinement

**Table:**

Refinement on *F*^2^	Primary atom site location: structure-invariant direct methods
Least-squares matrix: full	Secondary atom site location: difference Fourier map
*R*[*F*^2^ > 2σ(*F*^2^)] = 0.087	Hydrogen site location: inferred from neighbouring sites
*wR*(*F*^2^) = 0.109	H-atom parameters constrained
*S* = 1.12	*w* = 1/[σ^2^(*F*_o_^2^) + (0.025*P*)^2^] where *P* = (*F*_o_^2^ + 2*F*_c_^2^)/3
2558 reflections	(Δ/σ)_max_ < 0.001
190 parameters	Δρ_max_ = 0.16 e Å^−^^3^
1 restraint	Δρ_min_ = −0.15 e Å^−^^3^

### Special details

**Table:**

Geometry. All e.s.d.'s (except the e.s.d. in the dihedral angle between two l.s. planes) are estimated using the full covariance matrix. The cell e.s.d.'s are taken into account individually in the estimation of e.s.d.'s in distances, angles and torsion angles; correlations between e.s.d.'s in cell parameters are only used when they are defined by crystal symmetry. An approximate (isotropic) treatment of cell e.s.d.'s is used for estimating e.s.d.'s involving l.s. planes.
Refinement. Refinement of *F*^2^ against ALL reflections. The weighted *R*-factor *wR* and goodness of fit *S* are based on *F*^2^, conventional *R*-factors *R* are based on *F*, with *F* set to zero for negative *F*^2^. The threshold expression of *F*^2^ > σ(*F*^2^) is used only for calculating *R*-factors(gt) *etc*. and is not relevant to the choice of reflections for refinement. *R*-factors based on *F*^2^ are statistically about twice as large as those based on *F*, and *R*- factors based on ALL data will be even larger.

### Fractional atomic coordinates and isotropic or equivalent isotropic displacement parameters (Å^2^)

**Table:**

	*x*	*y*	*z*	*U*_iso_*/*U*_eq_	
O1	0.3661 (3)	0.90660 (11)	0.7245 (5)	0.0481 (8)	
O2	−0.0253 (3)	1.02162 (14)	0.7338 (6)	0.0901 (14)	
F1	0.0147 (4)	0.74844 (13)	0.3065 (5)	0.1002 (11)	
F2	0.1886 (3)	0.82036 (13)	0.2974 (4)	0.0759 (8)	
F3	−0.0440 (3)	0.83559 (15)	0.4155 (5)	0.0867 (10)	
C1	0.3889 (4)	0.96841 (17)	0.7160 (7)	0.0394 (10)	
C2	0.5488 (4)	0.98855 (19)	0.7098 (7)	0.0475 (12)	
H2	0.6339	0.9608	0.7105	0.057*	
C3	0.5801 (5)	1.04910 (19)	0.7029 (7)	0.0496 (12)	
H3	0.6872	1.0626	0.7023	0.060*	
C4	0.4543 (5)	1.09121 (19)	0.6967 (7)	0.0581 (13)	
H4	0.4767	1.1325	0.6878	0.070*	
C5	0.2973 (5)	1.07110 (18)	0.7040 (7)	0.0557 (13)	
H5	0.2132	1.0993	0.7028	0.067*	
C6	0.2595 (4)	1.00912 (18)	0.7131 (7)	0.0416 (11)	
C7	0.0917 (5)	0.98787 (19)	0.7367 (8)	0.0563 (14)	
C8	0.0756 (4)	0.92045 (17)	0.7675 (7)	0.0555 (15)	
H8A	−0.0302	0.9070	0.7254	0.067*	
H8B	0.0831	0.9119	0.8969	0.067*	
C9	0.2082 (4)	0.88567 (18)	0.6662 (6)	0.0393 (11)	
H9	0.1964	0.8925	0.5349	0.047*	
C10	0.2017 (4)	0.81852 (19)	0.7043 (6)	0.0365 (10)	
C11	0.1381 (5)	0.7759 (2)	0.5836 (6)	0.0387 (11)	
C12	0.1326 (5)	0.7148 (2)	0.6313 (7)	0.0478 (13)	
H12	0.0877	0.6868	0.5509	0.057*	
C13	0.1925 (5)	0.6955 (2)	0.7952 (8)	0.0540 (12)	
H13	0.1884	0.6545	0.8253	0.065*	
C14	0.2581 (5)	0.7360 (2)	0.9145 (7)	0.0583 (14)	
H14	0.2998	0.7227	1.0254	0.070*	
C15	0.2626 (5)	0.7976 (2)	0.8697 (7)	0.0505 (13)	
H15	0.3071	0.8252	0.9518	0.061*	
C16	0.0756 (6)	0.7942 (2)	0.4026 (8)	0.0594 (15)	

### Atomic displacement parameters (Å^2^)

**Table:**

	*U*^11^	*U*^22^	*U*^33^	*U*^12^	*U*^13^	*U*^23^
O1	0.0319 (14)	0.0360 (17)	0.076 (2)	−0.0008 (13)	−0.0020 (16)	0.001 (2)
O2	0.0468 (16)	0.064 (2)	0.160 (4)	0.0188 (17)	0.013 (2)	0.002 (3)
F1	0.149 (3)	0.079 (2)	0.072 (2)	−0.041 (2)	−0.051 (2)	−0.002 (2)
F2	0.0938 (18)	0.087 (2)	0.0464 (17)	−0.0169 (17)	0.0070 (17)	0.0086 (19)
F3	0.0697 (18)	0.097 (2)	0.093 (2)	0.0126 (19)	−0.0212 (18)	0.022 (2)
C1	0.044 (2)	0.032 (2)	0.042 (3)	−0.002 (2)	0.007 (2)	−0.009 (3)
C2	0.035 (2)	0.046 (3)	0.062 (3)	0.000 (2)	0.007 (3)	0.001 (3)
C3	0.050 (2)	0.045 (3)	0.055 (3)	−0.011 (2)	0.006 (3)	0.005 (3)
C4	0.070 (3)	0.031 (2)	0.073 (4)	−0.011 (3)	0.018 (3)	−0.005 (3)
C5	0.060 (3)	0.036 (3)	0.071 (3)	0.009 (2)	0.010 (3)	−0.001 (3)
C6	0.041 (2)	0.036 (3)	0.049 (3)	0.002 (2)	0.005 (3)	−0.002 (3)
C7	0.047 (2)	0.047 (3)	0.076 (4)	0.002 (2)	0.006 (3)	−0.013 (4)
C8	0.037 (2)	0.045 (3)	0.085 (4)	−0.002 (2)	0.009 (3)	−0.004 (3)
C9	0.040 (2)	0.042 (2)	0.036 (3)	−0.003 (2)	0.005 (2)	−0.007 (2)
C10	0.0344 (19)	0.037 (3)	0.038 (3)	−0.005 (2)	0.001 (2)	0.002 (3)
C11	0.030 (2)	0.044 (3)	0.042 (3)	−0.001 (2)	−0.002 (2)	0.002 (3)
C12	0.056 (3)	0.041 (3)	0.046 (3)	−0.001 (3)	0.004 (2)	−0.005 (3)
C13	0.060 (3)	0.035 (3)	0.067 (3)	−0.004 (3)	0.005 (3)	0.006 (3)
C14	0.066 (3)	0.060 (4)	0.050 (3)	−0.009 (3)	−0.014 (3)	0.015 (3)
C15	0.061 (3)	0.046 (3)	0.045 (3)	−0.008 (2)	−0.001 (3)	−0.006 (3)
C16	0.060 (3)	0.058 (4)	0.060 (4)	−0.011 (3)	−0.016 (3)	0.001 (4)

### Geometric parameters (Å, º)

**Table:**

O1—C1	1.376 (4)	C7—C8	1.508 (5)
O1—C9	1.443 (4)	C8—C9	1.526 (5)
O2—C7	1.217 (4)	C8—H8A	0.9700
F1—C16	1.328 (5)	C8—H8B	0.9700
F2—C16	1.339 (5)	C9—C10	1.506 (5)
F3—C16	1.345 (5)	C9—H9	0.9800
C1—C2	1.389 (5)	C10—C15	1.391 (5)
C1—C6	1.392 (5)	C10—C11	1.392 (5)
C2—C3	1.359 (5)	C11—C12	1.390 (6)
C2—H2	0.9300	C11—C16	1.480 (7)
C3—C4	1.390 (5)	C12—C13	1.367 (6)
C3—H3	0.9300	C12—H12	0.9300
C4—C5	1.367 (5)	C13—C14	1.361 (6)
C4—H4	0.9300	C13—H13	0.9300
C5—C6	1.401 (5)	C14—C15	1.397 (6)
C5—H5	0.9300	C14—H14	0.9300
C6—C7	1.468 (5)	C15—H15	0.9300
			
C1—O1—C9	115.2 (3)	O1—C9—C8	109.8 (3)
O1—C1—C2	116.5 (3)	C10—C9—C8	112.2 (3)
O1—C1—C6	122.3 (3)	O1—C9—H9	109.3
C2—C1—C6	121.2 (3)	C10—C9—H9	109.3
C3—C2—C1	119.6 (4)	C8—C9—H9	109.3
C3—C2—H2	120.2	C15—C10—C11	117.9 (4)
C1—C2—H2	120.2	C15—C10—C9	118.3 (4)
C2—C3—C4	121.0 (4)	C11—C10—C9	123.9 (4)
C2—C3—H3	119.5	C12—C11—C10	120.3 (4)
C4—C3—H3	119.5	C12—C11—C16	118.5 (4)
C5—C4—C3	119.1 (4)	C10—C11—C16	121.2 (4)
C5—C4—H4	120.4	C13—C12—C11	120.7 (5)
C3—C4—H4	120.4	C13—C12—H12	119.7
C4—C5—C6	121.8 (4)	C11—C12—H12	119.7
C4—C5—H5	119.1	C14—C13—C12	120.3 (5)
C6—C5—H5	119.1	C14—C13—H13	119.8
C1—C6—C5	117.3 (3)	C12—C13—H13	119.8
C1—C6—C7	120.8 (4)	C13—C14—C15	119.7 (5)
C5—C6—C7	121.6 (4)	C13—C14—H14	120.1
O2—C7—C6	123.2 (4)	C15—C14—H14	120.1
O2—C7—C8	122.3 (4)	C10—C15—C14	121.1 (4)
C6—C7—C8	114.5 (4)	C10—C15—H15	119.4
C7—C8—C9	111.0 (3)	C14—C15—H15	119.4
C7—C8—H8A	109.4	F1—C16—F2	106.4 (5)
C9—C8—H8A	109.4	F1—C16—F3	106.0 (4)
C7—C8—H8B	109.4	F2—C16—F3	104.9 (4)
C9—C8—H8B	109.4	F1—C16—C11	113.7 (4)
H8A—C8—H8B	108.0	F2—C16—C11	113.2 (4)
O1—C9—C10	106.9 (3)	F3—C16—C11	112.0 (5)
			
C9—O1—C1—C2	−158.8 (4)	C7—C8—C9—C10	176.3 (4)
C9—O1—C1—C6	21.0 (7)	O1—C9—C10—C15	43.2 (5)
O1—C1—C2—C3	−179.4 (5)	C8—C9—C10—C15	−77.3 (5)
C6—C1—C2—C3	0.7 (8)	O1—C9—C10—C11	−136.9 (4)
C1—C2—C3—C4	−1.8 (8)	C8—C9—C10—C11	102.7 (4)
C2—C3—C4—C5	2.2 (8)	C15—C10—C11—C12	1.7 (6)
C3—C4—C5—C6	−1.6 (8)	C9—C10—C11—C12	−178.2 (4)
O1—C1—C6—C5	−179.9 (4)	C15—C10—C11—C16	−178.1 (4)
C2—C1—C6—C5	−0.1 (7)	C9—C10—C11—C16	2.0 (6)
O1—C1—C6—C7	5.9 (7)	C10—C11—C12—C13	−1.4 (6)
C2—C1—C6—C7	−174.3 (5)	C16—C11—C12—C13	178.4 (4)
C4—C5—C6—C1	0.5 (8)	C11—C12—C13—C14	0.2 (7)
C4—C5—C6—C7	174.7 (5)	C12—C13—C14—C15	0.7 (7)
C1—C6—C7—O2	−179.4 (6)	C11—C10—C15—C14	−0.9 (6)
C5—C6—C7—O2	6.6 (9)	C9—C10—C15—C14	179.1 (4)
C1—C6—C7—C8	1.3 (7)	C13—C14—C15—C10	−0.3 (7)
C5—C6—C7—C8	−172.6 (5)	C12—C11—C16—F1	2.2 (7)
O2—C7—C8—C9	148.5 (5)	C10—C11—C16—F1	−178.0 (4)
C6—C7—C8—C9	−32.3 (6)	C12—C11—C16—F2	−119.4 (4)
C1—O1—C9—C10	−174.2 (4)	C10—C11—C16—F2	60.4 (6)
C1—O1—C9—C8	−52.2 (5)	C12—C11—C16—F3	122.3 (4)
C7—C8—C9—O1	57.6 (5)	C10—C11—C16—F3	−57.9 (6)

### Hydrogen-bond geometry (Å, º)

**Table:**

*D*—H···*A*	*D*—H	H···*A*	*D*···*A*	*D*—H···*A*
C9—H9···F2	0.98	2.36	3.068 (5)	129
C9—H9···F3	0.98	2.50	2.984 (5)	110
C12—H12···F1	0.93	2.33	2.677 (6)	102
C15—H15···O1	0.93	2.50	2.760 (5)	96
C3—H3···O2^i^	0.93	2.54	3.311 (5)	140
C5—H5···F3^ii^	0.93	2.54	3.313 (5)	141

Symmetry codes: (i) *x*+1, *y*, *z*; (ii) −*x*, −*y*+2, *z*+1/2.
